# Functional Diversity of the *Schistosoma mansoni* Tyrosine Kinases

**DOI:** 10.1155/2011/603290

**Published:** 2011-06-15

**Authors:** Lívia G. A. Avelar, Laila A. Nahum, Luiza F. Andrade, Guilherme Oliveira

**Affiliations:** ^1^Grupo de Genômica e Biologia Computacional, Centro de Pesquisas René Rachou, Fundação Oswaldo Cruz - FIOCRUZ, 30190-002 Belo Horizonte, MG, Brazil; ^2^Instituto de Ciências Biológicas, Universidade Federal de Minas Gerais - UFMG, 31270-910 Belo Horizonte, MG, Brazil; ^3^Instituto Nacional de Ciência e Tecnologia em Doenças Tropicais, Centro de Pesquisas René Rachou, Fundação Oswaldo Cruz - FIOCRUZ, 30190-002 Belo Horizonte, MG, Brazil; ^4^Centro de Excelência em Bioinformática, Fundação Oswaldo Cruz - FIOCRUZ, 30190-110 Belo Horizonte, MG, Brazil

## Abstract

*Schistosoma mansoni*, one of the causative agents of schistosomiasis, has a complex life cycle infecting over 200 million people worldwide. Such a successful and prolific parasite life cycle has been shown to be dependent on the adaptive interaction between the parasite and hosts. Tyrosine kinases (TKs) play a key role in signaling pathways as demonstrated by a large body of experimental work in eukaryotes. Furthermore, comparative genomics have allowed the identification of TK homologs and provided insights into the functional role of TKs in several biological systems. Finally, TK structural biology has provided a rational basis for obtaining selective inhibitors directed to the treatment of human diseases. This paper covers the important aspects of the phospho-tyrosine signaling network in *S. mansoni*, *Caenorhabditis elegans*, and humans, the main process of functional diversification of TKs, that is, protein-domain shuffling, and also discusses TKs as targets for the development of new anti-schistosome drugs.

## 1. Introduction

Blood flukes of the genus *Schistosoma* (Platyhelminthes: Trematoda) are the causative agents of schistosomiasis living in the bloodstream of their final hosts. Over 200 million people are infected worldwide and about 700 million live in endemic regions, with more than 90% of the cases of infection occurring in sub-Saharan Africa [1, 2].

Schistosomes have a complex developmental cycle with diverse life stages remarkably well adapted to their environment and hosts. Such a successful and prolific schistosome life cycle has been shown to be dependent on the adaptive molecular “dialogue” that takes place between the parasite and the host [3]. The contact of the parasite with host signals (e.g., hormones, growth factors, etc.) could control cell proliferation and differentiation processes in schistosomes [4]. In this context, diverse molecular pathways dependent on kinase-mediated signaling have been described in schistosomes and shown to be involved in host-parasite relationships [5].

Kinases play key roles in a broad range of cellular processes. The molecular phylogeny of the protein kinases upon which KinBase was developed [6] was first described by Hanks et al. [7]. In this classification scheme, the protein kinase superfamily is split into two groups: eukaryotic protein kinases (ePKs) and atypical protein kinases (aPKs). The former constitutes one of the largest and most important protein families in eukaryotes, accounting for ~2% of the total proteins identified in a variety of genomes [8]. The ePKs are further divided into nine groups (TK, AGC, CaMK, CMGC, CK1, STE, RGC, TKL, and other) based on sequence similarity of their catalytic domains, the presence of accessory domains, and their modes of regulation [6].

By phosphorylating substrate proteins, kinases modify the activity, location, and affinities of up to 30% of all cellular proteins and direct most cellular processes, particularly in signal transduction and coordination of complex pathways [8]. Many of these pathways are highly conserved among eukaryotes with 53 distinct kinase functions, which are conserved among yeasts, nematodes, insects, and vertebrates [8].

Multicellular life demands complex activities such as cell proliferation, differentiation, adhesion, and motility to be precisely controlled. Many of these processes are in fact regulated by protein tyrosine kinases (TKs). The tyrosine phosphorylation occurs* via *the covalent addition of a phosphate group from ATP or GTP to tyrosine residues in a variety of proteins, and the emergence of this signaling mechanism was likely a key enabling event in the transition to multicellularity [10].

The tyrosine kinase comprises cell surface receptor (RTK) and nonreceptor or cytosolic (CTK) kinases, classified into 29 families (Table 1). Deregulation of the tyrosine kinase activity by various mechanisms leading to gain or loss of function have been observed in a large number of tyrosine kinases and shown to be associated with different human diseases [11]. Due to their central regulatory roles, tyrosine kinases are considered interesting targets for the treatment of various diseases, most prominently cancer [12].

Recently, the draft genome of *S. mansoni *was published [13] and large-scale transcriptome projects have provided detailed information for the identification of protein kinases [14]. The ePK complement of *S. mansoni*, defined as the ePKinome, consists of 252 ePKs in the predicted proteome, representing 1.9% of the total proteins encoded the parasite genome [15]. Only 16 *S. mansoni* ePKs were experimentally studied, with 10 belonging to the TK group [16, 17].

The tyrosine kinases represent the fourth largest group of the *S. mansoni* ePKinome including 15 RTKs and 19 CTKs, classified into 18 families [15]. Of the parasite RTKs, 10 have homologs in six distinct human protein families, two belong to Venus kinase receptor family [18, 19], also present in many insects, and three were not grouped into families previously described in metazoans. At the moment, several tyrosine kinases characterized in *S. mansoni* are described as potential targets for therapy against schistosomiasis.

Tyrosine kinases constitute the largest group of ePKs in *Caenorhabditis elegans*, with 92 members, which represent 21% of all ePKs encoded in the nematode genome [20]. In* C. elegans*, these proteins correspond to 40 RTKs and 52 CTKs. The RTKs include 16 members of the worm-specific KIN-15-family, 13 RTKs with orthologs representing 10 of the 20 families of human RTKs, and 11 RTKs that remain unclassified with no identifiable mammalian counterpart [20].

The Fer family is the largest in *C. elegans*, with 37 members. Only a single member, SmFes, was observed in* S. mansoni* (Table 2). Furthermore, immunolocalization assays showed that SmFes is particularly expressed at the terebratorium of miracidia, an organ that helps the penetration of the parasite in the snail host, and tegument of cercaria and schistosomula skin stage [4]. These findings suggest that SmFes may play a role in signal transduction pathways involved in larval transformation after penetration into intermediate and definitive hosts [4, 15].

Ninety unique tyrosine kinase genes, representing ~17% of all ePKs, were identified in the human genome, along with nine pseudogenes [9, 21]. There were 58 RTKs distributed into 20 families and 32 CTKs grouped into 10 families [21].

Humans contain 14 members of Eph family, while only a single member (Smp_139480) was identified in* S. mansoni* (Table 2). Eph receptor signaling is responsible for the most diverse set of biological events performed by any tyrosine kinase including organ development, tissue remodeling, neuronal signaling and insulin secretion, and bone metabolism [16]. The *S. mansoni* Eph functional role remains unknown.

Here, we discuss the diversity of the *S. mansoni *tyrosine kinases from the functional and evolutionary perspectives. This review is organized in three main sections: phospho-tyrosine signaling network, tyrosine kinase functional diversification, and tyrosine kinases as new anti-schistosome drug targets.

## 2. Phosphotyrosine Signaling Network

**Figure 1:**
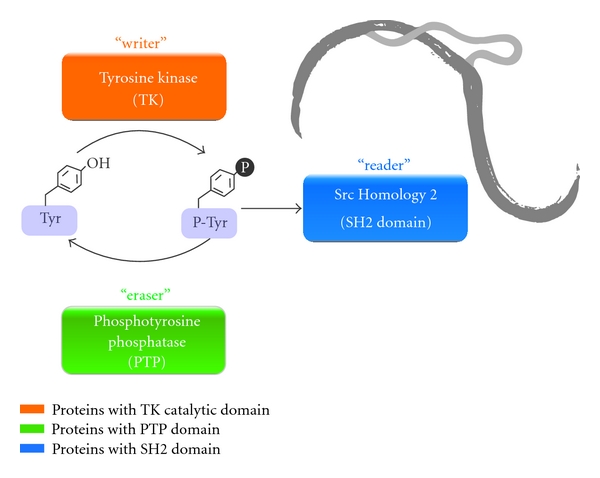
*S. mansoni* phosphotyrosine-signaling network. In the phosphotyrosine signaling pathway, the tyrosine kinase (TK), phosphotyrosine phosphatase (PTP), and Src Homology 2 (SH2) domains form a highly interdependent signaling network. At the moment, 81 protein members of the phosphotyrosine signaling network on *S. mansoni* genome were identified. This signaling network serves as the “*writer*”, “*eraser*”, and “*reader*” domains, respectively, for processing phosphotyrosine targets.

The cellular signaling machinery mediated by tyrosine kinases is widely studied in modern metazoans [22]. In these organisms phosphotyrosine-signaling pathways are mediated by a “*toolkit*” of three functional protein domains (Figure 1): the tyrosine kinase catalytic domain (TK) that phosphorylates-specific target tyrosine residues, the phosphotyrosine-phosphatase domain (PTP) that removes the phosphates, and the Src Homology 2 domain (SH2) that recognize these modifications [23]. Together, these domains form the “*writer*,” “*eraser*,” and “*reader*” domains that is common to many diverse cellular information processes [24]. All members of phosphotyrosine network are found in *S. mansoni*.

Phosphotyrosine-binding (PTB) domains also participate in tyrosine kinase-signaling networks (not included in Figure 1). According to the *S. mansoni* relational database, *SchistoDB* (www.schistodb.net) [14], there are two genes, Smp_139400 and Smp_126500, that code for proteins with significant similarity to the PTB domain (PF08416) as defined by the Pfam database [25]. The *S. mansoni* PTB domains are members of the Tensin cytoplasmic phospho-protein (Tec) family. PTB domains are underrepresented in the *S. mansoni* genome when compared to the 37 proteins with SH2 domains (Figure 1, “*reader*”). There are nine tyrosine kinases among the SH2-containing domain proteins in *S. mansoni*. The remaining ones are tensin, suppressors of cytokine signaling, Ty suppressors, Rho GTPase, Ras GTPase, and adaptor proteins.

Analysis of the *C. elegans *genome has indicated 11 PTB domain proteins related to the phosphotyrosine-binding activity [26]. Similar to *S. mansoni*, the PTB domain is relatively underrepresented when compared to the 57 proteins with SH2 domains encoded in the *C. elegans *genome [27].

There are nearly 60 PTB domain proteins in humans [28], six of which have been reported to carry mutations that contribute to inherited human diseases such as familial stroke, hypercholesteremia, coronary artery disease, Alzheimer's disease, and diabetes [24]. These findings demonstrated that these proteins play an important role in organizing signaling complexes in a broad range of physiological processes [24]. PTB domains also bind head groups of acidic phospholipids consistent with the nearly exclusive subcellular localization of PTB domains to the membrane or juxtamembrane regions, suggesting that most PTB domains are multifunctional [29]. Similar to *S. mansoni* and *C. elegans*, the PTB domain is relatively underrepresented when compared to the 110 proteins with SH2 domains encoded in the human genome.

The number of PTB domain proteins is greater in human than in *S. mansoni* and *C. elegans* proteomes [20]. In addition, among the 946 PTB domain proteins deposited in SMART [30], eight are found in echinoderms, 28 in nematodes, 58 in arthropods, and 852 in chordates. These data suggest higher levels of diversity of the vertebrate PTB domains in relation to invertebrates.

Searching the *S. mansoni* relational database, *SchistoDB* [14], for proteins with significant similarity to the tyrosine phosphatase sequence domain (PF00102) (Figure 1, “*eraser*”) as defined by Finn et al. [25], there are 18 genes potentially encoding tyrosine phosphatases in this parasite.

The number of tyrosine kinase encoding genes (34 proteins) in the *S. mansoni* genome is higher than that of tyrosine phosphatases, which suggests that these enzymes may act on different substrates. However, we should consider the following observations: (1) among the 34 *S. mansoni* tyrosine kinases, four are predicted to be catalytically inactive, while the number of inactive tyrosine phosphatases is not known at the moment; (2) there are eight genes encoding members of the dual specificity phosphatase family, which can dephosphorylate both phosphotyrosine and phosphoserine or phosphothreonine residues within one substrate; (3) the *S. mansoni* genomic data remains fragmented and much work is still necessary to complete the assembly of the genome sequences [31]. Therefore, the number of tyrosine phosphatases may be underestimated in the actual assembly and annotation of the *S. mansoni* genome.

Analysis of the *C. elegans *genome identified 91 tyrosine phosphatase genes [32]. Generally, worms contain a similar number of tyrosine kinases and phosphatases. This coordinate expansion in the nematode lineage could possibly reflect the biological need to maintain a tight regulation of the phosphotyrosine process.

The human genome encodes 107 tyrosine phosphatase family members [33], which together exceed the number tyrosine kinases in the same organism [8]. However, a more detailed inspection reveals that only 81 proteins are active phosphatases with the ability to dephosphorylate phosphotyrosine residues. The remaining phosphatases are catalytically inactive (11 proteins), dephosphorylate mRNAs (two proteins), or dephosphorylate inositol phospholipids (13 proteins). Out of the 90 human tyrosine kinases, 85 are believed to be catalytically active leading to similar numbers of active tyrosine phosphatases and kinases in humans. Furthermore, both enzyme types display comparable patterns of tissue distribution [33].

Recently, the crucial role of tyrosine phosphorylation was shown in snail-schistosome interactions [34]. The exposure of miracidia to the haemolymph of schistosome-susceptible snails is followed by increased protein tyrosine phosphorylation profile. In addition, the treatment of miracidia with a tyrosine kinase-specific inhibitor significantly impaired their development into primary sporocysts. These results suggest the participation of signal transduction pathways mediated by tyrosine kinases during the snail-host infection and transformation of the evolutionary stages of the *S. mansoni* life cycle.

*Schistosoma* proteins SmTK3 (Smp_054500) and SmTK5 (Smp_136300) are Src family members, while SmTK4 (Smp_149460) belongs to the Syk family. The later is present in reproductive organs and it is possibly involved in the development of gonads and oogenesis [36, 37].

Detailed knowledge of the signaling pathways that control schistosome growth, metabolism, differentiation and survival is of particular interest because only mature adult worms produce eggs, which are responsible for disease pathology.

## 3. Tyrosine Kinase Functional Diversification

**Figure 2:**
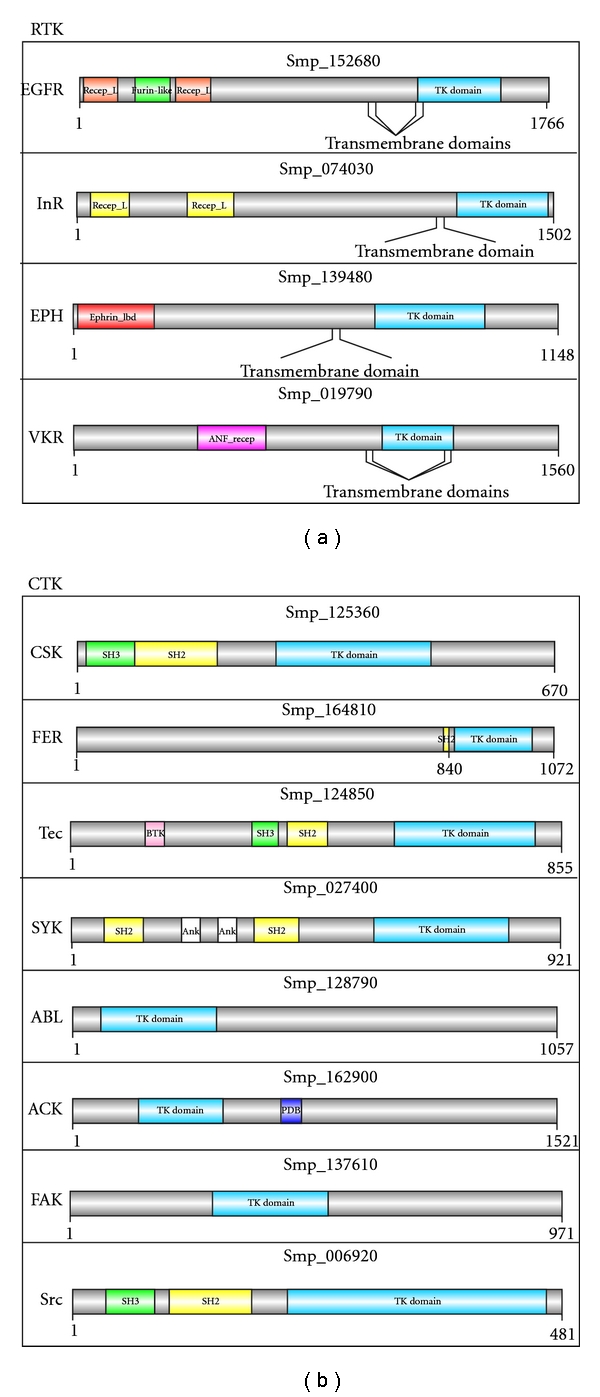
Representative sequence domain architectures of some *S. mansoni *TKs belonging to the receptor and nonreceptor members. Protein family abbreviation is indicated on the left side of each protein architecture (see the list of abbreviations for the respective full names). Protein identifiers (e.g., Smp_125360) shown above each image were retrieved from *SchistoDB* [14]. Abbreviations followed are: PTK_domain (protein tyrosine kinase catalytic domain), SH2 (Src Homology 2 domain), SH3 (Src Homology 3 domain), PDB (P21-Rho-binding domain), Ank (ankyrin repeat), Recep_L_domain (receptor L domain), Furin-like (furin-like cysteine rich region), ANF_recep (receptor family ligand-binding region), Ephrin_Ibd (ephrin receptor ligand-binding domain), and BTK (Bruton's tyrosine kinase motif). The protein domain architectures were generated using DOG 1.0 [35] based on the domain limits Pfam [25].

A simple way to assess molecular diversity of gene/protein families is to analyze their domain organization [38, 39]. The functional diversity of the* S. mansoni* tyrosine kinases is reflected by the presence of 14 distinct accessory domains besides the catalytic domain that is found in all ePKs (Figure 2). Following the tyrosine kinase catalytic domain, the two most frequently occurring protein domains in the *S. mansoni* tyrosine kinases are Src Homology 2 (SH2) and Src Homology 3 (SH3) domains.

Important clues regarding the relationships between SH2 and SH3 domains are provided by the genomes of unicellular eukaryotes, which lack the complete set of phosphotyrosine signaling machinery [26].

The genome of a simple unicellular eukaryote like the budding yeast, *Saccharomyces cerevisiae* shows one proto-SH2 domain, which shares similarity with the SH2 domain from other organisms but, does not show the phosphotyrosine-binding activity [40]. Although SH2 is not present in prokaryotes [41], a variety of SH2-containing tyrosine kinases have been found in organisms, such as the sponge, implying that many of the domain rearrangements happened early in metazoan evolution [30]. Tyrosine phosphorylation mediates the formation of heteromeric protein complexes at or near the plasma membrane by acting as a “*switch*” to induce the SH2 domain binding as described elsewhere [30]. The formation of these protein complexes, on the other hand, is likely to control the activation of signal transduction pathways by tyrosine kinases. Thus, the SH2 domain serves as the prototype for a growing family of protein-interaction domains, characteristic of polypeptides involved in signal transduction pathways. Together with the SH2 domain, the SH3 may modulate interactions with the cytoskeleton and membrane.

The SH2 domain is present in eight *S. mansoni *tyrosine kinases grouped into four CTK families: Csk, Fer, Scr, Syk, and Tec (Figure 2). Members of Scr and Syk families have been characterized and are involved in organizing the cytoskeleton in the parasite gonads [42] and germ cell development [37].

The six SH3-containing domain tyrosine kinases in *S. mansoni* are present in three CTK families: Csk, Syk, and Tec (Figure 2). Members of Csk and Syk families have the SH3 domain in addition to SH2 and the tyrosine kinase catalytic domains. In addition to these domains, members of the Tec family contain two other domains: Bruton's tyrosine kinase (BTK) and Pleckstrin homology (PH) domains. Similar domain architecture is observed in the Tec family of the choanoflagellate,* Monosiga brevicollis*. Currently, *M. brevicollis* is the only unicellular organism that presents a tyrosine kinase signalling network that has been either characterized experimentally or identified by computational prediction [26].

An alternative way to investigate functional diversification of proteins and protein families is through phylogenomics (“intersection between *phylo*genetics and *genomics*”) as previously proposed [43]. This evolutionary framework, originally designed to improve functional prediction of uncharacterized genes/proteins, has been applied to a broad range of studies [43, 44].

The relationships among 23 selected tyrosine kinases from *S. mansoni* were inferred by phylogenomic analysis of their catalytic domain sequence (data not shown). Tree information corroborates the grouping of these proteins into distinct families encoded in the parasite genome, such as EGFR (epidermal growth factor receptor) and VKR (venus flytrap kinase receptors).

As mentioned before, most of these proteins remain experimentally uncharacterized. Some of them are proposed as drug targets, that is, ABL and EGFR family members. The aforementioned approach could be used as a framework for hypothesis testing to gain insights into the changes leading to sequence and functional diversification across proteins/organisms over evolutionary time.

## 4. Tyrosine Kinases as New Anti-schistosome Drug Targets

In the past decades, “a single drug for a single target” paradigm has dominated drug discovery approaches. A systems-biology approach, especially focused on the elucidation of cellular signaling pathways, could provide a framework, for anti-schistosome drug discovery [45].

Signaling pathways controlled by protein kinases are a central theme in biological systems. An aberrant protein kinase activity has been implicated in a variety of human diseases, such as cancer, rheumatoid arthritis, and cardiovascular and neurological disorders [46]. Therefore, modulation of kinase activity represents an attractive therapeutic approach for human diseases. The design and development of specific inhibitors for protein kinases, thus, became a major strategy in many drug discovery programs [11].

When the focus is the discovery of new drugs against schistosomiasis it is necessary to answer some key questions: What is the current need for new drugs against schistosomiasis? What are the challenges faced in the process of finding drugs? What genes/proteins can be used as potential chemotherapeutic targets? And finally, are tyrosine kinases potential targets for new drugs against* Schistosoma* species?

The drug Praziquantel (PZQ), for which the detailed mode of action is still unclear [47], is the only commercially available treatment for the schistosomiasis. PZQ success as a drug has contributed to a lack of urgency and investment in identifying new therapies, either in terms of searching for chemical entities or molecular targets. However, resistance to PZQ has been developed in more than one occasion in the laboratory [48], and the extensive use of PZQ in mass drug administration programs has raised concern regarding the selection of drug resistant schistosomes in the field [47].

There has been little incentive to invest in the discovery and development of antitrematode drugs. However, public-private partnerships have been formed for some of the neglected tropical diseases. One example of such partnership is the Drugs for Neglected Diseases Initiative (DNDi), focusing on human African trypanosomiasis and leishmaniasis. Drug discovery and development programs do not yet exist for any of the major helminthoses such as schistosomiasis [49].

By using a comparative chemogenomics approach, Caffrey and colleagues have identified 72 potential target proteins in the *S. mansoni* predicted proteome [50]. Among the 72 proteins identified, two are protein kinases grouped into the GSK and CMGC families. Furthermore, some anticancer drugs developed to inhibit deregulated protein kinases can also inhibit schistosome enzymes, thus blocking parasite development [51].

Polo kinases (Plks) have crucial conserved functions in controlling the eukaryotic cell cycle through several events during mitosis [52]. *S. mansoni* Polo kinase, SmPlk1, was identified and characterized [53]. Using the specific inhibitor, BI 2536, to block SmPlk1 kinase activity caused profound alterations in the gonads of both genders, including a reduction of gamete production. At present, the dihydropteridinone compound BI 2536 is the most potent and advanced anti-Plk1 molecule in clinical trials [18].

Eight PK inhibitors with anticancer properties display activities on schistosomes. Tyrphostins AG 538 and AG 1024 [54] as well as HNMPA-(AM) 3 [55] inhibit the tyrosine kinase of human insulin receptors and were shown to reduce glucose uptake in schistosomes. Both the TGF*β*-R tyrosine kinase inhibitor TRIKI and the Src-kinase inhibitor Herbimycin A reduce mitotic activity and fecundity in schistosomes in an additive manner [56]. H89, an inhibitor of the catalytic unit of PKA (PKA-C), induces loss of egg production and viability in schistosomes [57]. Piceatannol, an inhibitor of the Syk tyrosine kinase, provokes reduction of egg production in treated schistosomes [55]. Imatinib, an Abl kinase inhibitor, approved by the US Food and Drug Administration (FDA) and used in the clinic, has fatal physiological effects on schistosomes *in vitro* at doses similar to those used for cancer treatment in humans [51, 58]. Treatment with Imatinib is generally well tolerated, even over a period of many years, with a low incidence of severe side-effects.

The results obtained using anticancer drugs for treating schistosomiasis suggest that the treatment period using a kinase inhibitor with schistosome-killing properties will be significantly shorter compared to cancer treatment [51]. However, three main points make it difficult to use kinase inhibitors as anti-schistosome compound when compared to PZQ: (1) unlike most kinase inhibitors, PZQ is generally well tolerated; (2) PZQ represents a low-cost medicine, whereas cancer drugs such as Imatinib are still expensive, and (3) in particular, protein kinases share very similar structural and functional features, making it more difficult to design a specific inhibitor.

Issues regarding the enzyme specificity may be overcome by drug redesign guided by the identification of structural features that promote promiscuity and selectivity filters that enable target discrimination [59]. Furthermore, costs could be brought down* via *generic producers with expiring patents, which in the case of Imatinib will happen soon. Second-generation compounds for the same target are already in the pipeline, and this will have an additional effect on price reduction [51].

The use of TK inhibitors as chemotherapeutic agents may be extended to other helminth parasites, including the filarial nematode *Brugia malayi*. Indeed, the genome of this parasite encodes a total of 205 protein kinases that are potential drug targets and correspond to about half of the human complement [60].

Tyrosine kinases have been shown to be essential for the proliferation and/or viability of clinically relevant schistosome life-cycle stages [53]. Moreover, the available data concerning the expression of tyrosine kinases throughout the life-cycle, which are accessible at *SchistoDB* [14], show that a number of these enzymes are expressed in schistosomula and/or adult worms, the therapeutic targets. Serial analysis of gene expression (SAGE) data shows that 19 tyrosine kinases are expressed in adult worms (Table 3). On the other hand, expressed sequence tag (EST) suggests that 18 are expressed in adult worms, among which 11 are also expressed by 3-day old schistosomula and one by 7-day-old schistosomula. One CTK (Smp_134800) is expressed only in 7-day-old schistosomula (Table 3). Quantitative real-time PCR assays are also necessary to verify stage specificity of tyrosine kinases expression. However, TKs expressed in common by adult worms and schistosomula should probably be preferentially targeted for drug discovery. 

Therefore, tyrosine kinases inhibitors are applicable to the development of alternative strategies to reduce both pathology and transmission of schistosomiasis [61].

## 5. Conclusion and Future Perspectives

Schistosome studies have truly entered a new stage with the recent publication of the *S. mansoni* [13, 62] and *S. japonicum* genomic sequence data [63]. It is now vital to investigate the functional roles of gene products to answer questions concerning the fundamental biology of these important human parasites. As discussed here, tyrosine kinases, which participate in signaling pathways, are of interest when it comes to understand organisms such as parasites.

Multicellular organisms use a, three-protein domain, “*toolkit*” to mediate phosphotyrosine signaling: tyrosine kinases catalytic (“*writer*”), tyrosine phosphatase catalytic (“*eraser*”), and Src Homology 2 (“*reader*”) responsible for phosphotyrosine modifications of a variety of proteins [39]. Phosphotyrosine signaling is a complex system that exerts crucial biological effects by regulation of interactions at the molecular and physiological levels. All members of phosphotyrosine machinery were found in *S. mansoni*. The CCK4, FAK, Musk, SYK, and Tec tyrosine kinase families are found both in *S. mansoni* and in humans, but not in *C. elegans* (Table 2). The VKR family is present neither in *C. elegans* nor in humans, nor in the model insect *Drosophila melanogaster* [18]. Moreover, *S. mansoni* is the only organism so far discovered in which more than one representative of the VKR family is present.

Domain shuffling has been observed in several organisms leading to sequence, structural, and/or functional diversification of proteins [64]. The functional diversity observed in the* S. mansoni* tyrosine kinases is reflected by the presence and distinct combinations of 14 accessory domains besides the catalytic domain, which is found in all ePKs described so far. *S. mansoni* has a complex life cycle; therefore, acquiring proteins with new functions is essential for the evolution of the parasite.

The dependence on a single drug, PZQ, for treating schistosomiasis and the reports of possible resistance [48] motivates the search for new drug targets. The design and development of specific inhibitors for tyrosine kinases thus have became a major strategy in many drug discovery programs [11]. Tyrosine kinases have been shown to be essential for proliferation and/or viability of parasite life-cycle stages that are clinically relevant [51]. Therefore, tyrosine kinases inhibitors are applicable to the development of alternative strategies to reduce both pathology and transmission of schistosomiasis.

Combining computational and experimental approaches of other helminth parasites, whose genome sequencing projects are underway, should greatly advance our understanding on the functional diversity of tyrosine kinases and the parasite and on the parasite biology and evolution.

## Figures and Tables

**Table 1:** Protein tyrosine kinase classification. Tyrosine kinases classified into ePK groups, families, and subfamilies followed the proposed hierarchy described elsewhere [7–9]. RTK: receptor tyrosine kinase. CTK: cytoplasmic tyrosine kinase.

Type	Abbreviation	Family name
RTK	Alk	Anaplastic lymphoma kinase
	Axl	Also known as TAM (Tyro3, Axl, Mer) after the three human members
	CCK4	Colon carcinoma Kinase 4
	DDR	Discoidin domain receptor kinase
	EGFR	Epidermal growth factor receptor
	Eph	Ephrin receptor
	FGFR	Fibroblast growth factor receptor
	InR	Insulin Receptor
	Met	MET or MNNG HOS transforming gene
	MUSK	Muscle-specific kinase
	PDGFR	Platelet-derived growth factor receptor
	Ret	Ret proto-oncogene
	ROR	RAR-related orphan receptor
	Tie	Tyrosine kinase with immunoglobulin-like and EGF-like domains
	VEGFR	Vascular endothelial growth factor receptor
	VKR	Venus flytrap kinase receptor

CTK	Abl	Abelson murine leukemia homolog
	Ack	Activated Cdc42-associated tyrosine kinase
	Csk	Src subgroup kinase which phosphorylates Src
	Fak	Focal adhesion kinase
	Fer	Fps/Fes related
	Jak	Janus kinase
	Lmr	Lemur kinase
	Ryk	Rich protein kinase
	Sev	Named after *Drosophila* sevenless, a receptor tyrosine kinase involved in eye cell fate determination
	Src	v-Src sarcoma viral oncogene homolog
	Syc	Syc protein
	Tec	Tec protein tyrosine kinase
	Trk	Trk protein kinase

**Table 2:** Distribution of some tyrosine kinase families in *S. mansoni*, *C. elegans*, and human. *S. mansoni* tyrosine kinases were classified according to KinBase [6] by combining sequence similarity searches (HMMs) and phylogenetic analysis [15]. For comparison, occurrence of the ePKs families in *C. elegans* and human is shown. RTK: receptor tyrosine kinase. CTK: cytoplasmic tyrosine kinase. (See the list of abbreviations for the respective family full name).

Type	Family	*S. mansoni*	*C. elegans*	*H. sapiens*
RTK	CCK4	1	0	1
	EGFR	4	1	4
	Eph	1	1	14
	InsR	2	1	3
	Musk	1	0	1
	Ror	1	1	2
	VKR	2	0	0

CTK	Abl	2	1	2
	Ack	2	2	2
	Csk	1	1	2
	Fak	1	0	2
	Fer	1	37	2
	Ryk	1	1	1
	Sev	1	1	1
	Src	6	3	11
	SYK	2	0	2
	Tec	1	0	5
	Trk	1	1	3

**Table 3:** EST and SAGE data of the *S. mansoni *tyrosine kinases. Protein identifiers were retrieved from *SchistoDB*. [14]. Gene expression was evaluated in three different mammalian host stages, 3- and 7-day-old schistosomula and adult worms. RTK: receptor tyrosine kinase. CTK: cytoplasmic tyrosine kinase. Protein families are also indicated. (See the list of abbreviations for the respective family full name).

Type	*SchistoDB*	Family	3-day schistosomula	7-day schistosomula	Adult worms	
			EST	EST	EST	SAGE
RTK	Smp_173380	CCK4	X		X	
	Smp_093930.2	EGFR	X		X	X
	Smp_152680	EGFR				X
	Smp_165470	EGFR			X	X
	Smp_173590	EGFR	X		X	X
	Smp_139480	Eph				X
	Smp_009990	InsR	X		X	X
	Smp_074030	InsR				X
	Smp_136550	Musk			X	
	Smp_019790	VKR	X		X	X
	Smp_153500	VKR				X

CTK	Smp_169230	Abl				X
	Smp_128790	Abl	X		X	X
	Smp_162900	Ack	X		X	X
	Smp_164930	Ack	X	X		
	Smp_125360	Csk		X	X	X
	Smp_137610	Fak			X	X
	Smp_164810	Fer			X	X
	Smp_006920	Src			X	
	Smp_054500	Src	X		X	
	Smp_136300	Src	X		X	X
	Smp_027400	SYK	X		X	X
	Smp_149460	SYK	X		X	X
	Smp_124850	Tec			X	X
	Smp_134800	TRK		X		

## Abbreviations

Abl: Abelson murine leukemia homolog
Ack: Activated Cdc42-associated tyrosine kinase
AGC: cAMP-dependent protein kinase/protein kinase G/protein kinase C extended
Ank: Ankyrin repeat
aPKs: Atypical protein kinases
AXL: Anexelekto, uncontrolled, transforming gene in chronic myelogenous leukaemia
BTK: Bruton's tyrosine kinase motif
CAMK: Calcium/Calmodulin regulated kinases
CaMK2: CaMK family 2
CK1: Cell kinase I
CMGC: Cyclin-dependent kinases and other close relatives
CSK: C-src tyrosine *kinase *
CTK: Cytoplasmatic tyrosine kinase
DNDi: Drugs for neglected diseases initiative
EGFR: Epidermal growth factor receptor
Ephrin_Ibd: Ephrin receptors ligand-binding domain
EPH: Ephrin receptor
ePKs: Eukaryotic protein kinases
FAK: Focal adhesion kinase
FDA: Food and Drug Administration
Fer: Fps/Fes related
FGFR: Fibroblast Growth Factor Receptor
Furin-like: Furin-like cysteine-rich region
GTP: Guanosine triphosphate
HNMPA-(AM)3: Hydroxy-2-naphthalenylmethylphosphonic acid tris acetoxymethyl ester
InsR: Insulin Receptor
Jak: Janus kinase
Lmr: Lemur kinase
Met: Methyl-nitroso-nitroguanidine-induced oncogene
mRNA: Messenger RNA
PDGFR: Platelet-derived growth factor receptor
Pfam: Protein families database
PK_domain: Protein kinase domain
PKA: Protein Kinase A
PKC: Protein Kinase C
PK: Protein kinase
Plk: Polo kinases
PTB: Phosphotyrosine binding
PTK_domain: Protein tyrosine-kinase domain
PTP: Phosphotyrosine-phosphatase domain
PZQ: Praziquantel
Ras: Rat sarcoma
Recep_L_domain: Receptor L domain
Ret: Rearranged during transfection
RGC: Receptor guanylate cyclases
Rho: Ras homolog
Ror: Orphan receptor
RTK: Receptor tyrosine kinase
SH2: Src Homology 2 domain
SH3: Src Homology 3 domain
STE: MAP kinase cascade kinases
Syk: Spleen tyrosine kinase
TGFßR: Transforming growth factor beta receptor
TK: Protein tyrosine kinase
TKL: Tyrosine kinase like
VKR: Venus flytrap kinase receptors.
